# Examining variations in the prevalence of hazardous opioid prescribing across general practices in England: a cross-sectional study

**DOI:** 10.1136/bmjqs-2025-018794

**Published:** 2025-08-05

**Authors:** Teng-Chou Chen, Alex M Trafford, Matthew J Carr, Neetu Bansal, Evangelos Kontopantelis, Anthony Avery, Li-Chia Chen, Darren M Ashcroft

**Affiliations:** 1 Department of Pharmacy, School of Pharmaceutical Sciences, National Yang Ming Chiao Tung University, Taipei, Taiwan; 2 Division of Pharmacy and Optometry, School of Health Sciences, Faculty of Biology, Medicine and Health, University of Manchester, Manchester, UK; 3 National Institute for Health and Care Research Greater Manchester Patient Safety Research Collaboration, Manchester Academic Health Science Centre, University of Manchester, Manchester, UK; 4 Division of Informatics, Imaging and Data Sciences, University of Manchester, Manchester, UK; 5 School of Medicine, University of Nottingham, Nottingham, UK; 6 National Institute for Health and Care Research Manchester Biomedical Research Centre, Manchester Academic Health Science Centre, University of Manchester, Manchester, UK

**Keywords:** England, general practice, medication safety, anaesthesia

## Abstract

**ABSTRACT:**

**Background:**

Prescribed opioids are potent analgesics associated with high safety risks due to their adverse effects, drug-drug and drug-disease interactions and potential for dependency. To support the implementation of prescribing indicators for further interventions, this study examined the prevalence of different types of potentially hazardous opioid prescribing (PHOP) in general practices across England and investigated underlying factors and variation between practices.

**Methods:**

We conducted a cross-sectional study focusing on adults (aged ≥18 years) at risk of triggering 17 PHOP indicators on 1 April 2021, involving 1358 general practices contributing to the Clinical Practice Research Datalink Aurum. PHOP prevalence was calculated by dividing the number of patients triggering an indicator by the total number at risk. Variation was assessed with intraclass correlation coefficients (ICCs), and multilevel mixed-effects logistic regression models identified associated factors, presented as adjusted ORs (aORs) with 95% CIs.

**Results:**

Among 3 121 852 patients observed, 361 505 (11.58%, 95% CI 11.54, 11.62) triggered at least one PHOP indicator, yielding an ICC of 0.07 (95% CI 0.06, 0.07). The prevalence of the 17 PHOP indicators ranged from 1.97% to 32.02%. Significant variability was noted across the 17 indicators, especially for persistent opioid prescriptions in patients with alcohol use issues (ICC 0.08, 95% CI 0.07, 0.09), chronic obstructive pulmonary disease or asthma (ICC 0.08, 95% CI 0.07, 0.09) and hypothyroidism (ICC 0.07, 95% CI 0.06, 0.07). Patients from the most deprived regions (aOR 1.28, 95% CI 1.22, 1.34) and the Northwest of England (aOR 1.73, 95% CI 1.66, 1.81) had a higher risk of PHOP.

**Conclusions and relevance:**

The high prevalence of PHOP, particularly among the most socioeconomically disadvantaged populations, emphasises existing prescribing risks and the need for their appropriate consideration within primary care. The high variation between practices indicates potential for improvement through targeted practice-level intervention.

**WHAT IS ALREADY KNOWN ON THIS TOPIC**

**WHAT THIS STUDY ADDS**

- In a broadly representative sample of 1358 general practices in England, about 10% of adult patients at risk were found to have received potentially hazardous opioid prescribing, especially for patients residing in the most deprived areas.
- Indicators of persistent prescription of opioids to patients with alcohol use issues, hypothyroidism and respiratory diseases had the highest between-practice variation, even after adjustment for patient and practice-level characteristics, pointing towards important targets for improving medication safety in primary care.

**HOW THIS STUDY MIGHT AFFECT RESEARCH, PRACTICE OR POLICY**

- The high variation between practices indicates potential for improvement through targeted practicelevel intervention.
- Further studies should identify the feasibility of implementing opioid prescribing indicators with higher between-practice variation in more deprived areas to reduce hazardous opioid use.

## Background

 The rise in opioid prescribing in the UK, coupled with increasing opioid-related harms and mortality from 22.9 to 43.8 cases per million people from 2012 to 2023,^1^ underscores the need for increased investment in chronic pain management.^2^ Opioid analgesics have traditionally been used for managing acute and cancer-related pain as recommended by the WHO analgesic ladder.^3^ However, opioids are potent analgesics associated with high safety risks due to their adverse effects, drug-drug and drug-disease interactions and potential for dependency.^4^

Over the past two decades, there has been a troubling surge in opioid prescriptions within UK primary care for chronic non-cancer pain management.^5 6^ Although the rate of opioid prescribing decreased from 2012 in the UK, the prescribing of high-dose and long-acting opioids increased by around 460% up to 2018.^7 8^ From 2017 to 2018, approximately 12% of the UK population had received at least one opioid prescription,^9^ with 1.1 million patients being prescribed opioids over a year, thus heightening their risk of adverse drug events.^10^ In the UK, adverse drug events account for 7% of all hospital admissions, with over half of these incidents being preventable.^11 12^ Opioid-related hospitalisations increased by almost 50%, from 10 805 admissions in 2008 to 16 091 in 2018 in England.^13^

Persistent and high-dose opioid use (at oral morphine equivalent (OMEQ) doses exceeding 120 mg/day) correlates with an increasing risk of opioid-related harm.^14 15^ Furthermore, many patients with chronic non-cancer pain present with comorbidities (such as depression) or are prescribed multiple medications for pain management, further complicating their risk profile for drug-drug and drug-disease interactions.^16 17^

Current guidelines advise against persistent prescribing due to the lack of robust evidence supporting the effectiveness of long-term opioid use for patients with chronic non-cancer pain.^10 18 19^ Public Health England has acknowledged these concerns in its review of the benefits and dependence risks associated with opioid analgesics.^10^ The National Institute for Health and Care Excellence has also issued guidance on optimising medicines for chronic pain in 2017, alongside resources like the ‘Opioid aware’ guide by the Faculty of Pain Medicine to support healthcare professionals and patients in navigating opioid prescriptions.^20 21^ Despite these efforts, a cross-sectional study reported that around 47% of patients with chronic pain were still prescribed opioids due to the difficulty in accessing and engaging non-pharmacological therapy,^4 22^ and the implementation of these guidelines into routine clinical practice remains challenging. A systematic approach is crucial to mitigate potential harm and enhance patient safety effectively.^23^

Prescribing safety indicators refer to measurable, predefined prescribing patterns that signal an increased risk of harm and should generally be avoided.^24^ Prescribing safety indicators could be implemented in various types of interventions, including educational guidance, a prescribing dashboard or financial incentives, to reduce inappropriate prescribing and improve the quality of care.^25^

To optimise opioid prescribing for patients with chronic non-cancer pain, we previously developed a suite of opioid prescribing safety indicators based on a literature review and Delphi expert consensus.^26^ However, the prevalence and utility of potentially hazardous opioid prescribing (PHOP), and variations between general practices have not yet been explored. In addition, those indicators of opioid prescribing were not absolutely contraindicated, and some exceptions to prescribing indicators could be justified for clinical reasons. For example, patients with chronic pain may benefit from prescribing of antidepressants, but it is important to recognise prescribing of central nervous system depressants with opioids could increase the risk of respiratory depression.

Therefore, to advance the development of these indicators as part of an effective translational pathway to implementation, it is crucial to set a benchmark and identify the target population for further application to mitigate PHOP within primary care settings.^27^ This study aims to identify variations in the prevalence of PHOP across general practices and its associated factors. The objectives are to: (1) assess the prevalence of PHOP in primary care; (2) explore variation in PHOP between general practices; (3) examine patients’ and practices’ characteristics associated with the occurrence of PHOP.

## Methods

### Study design and data sources

This cross-sectional study examined primary care electronic health records from the Clinical Practice Research Datalink (CPRD) Aurum, linked to the practice-level Index of Multiple Deprivation (IMD) based on the location of the 1358 practices.^28^ CPRD has been found to be representative of the UK population regarding sex, age and ethnicity distributions, providing longitudinal clinical records from routine practice that enable the comprehensive identification of patients’ comorbidities and prescriptions.^28^ This study followed the REporting of studies Conducted using Observational Routinely collected health Data guidelines.^29^

### Study cohort

Patients registered in practices that contributed data to CPRD Aurum up to 1 April 2021 (audit date) were included. Individuals younger than 18 years and those with <1 year of up-to-standard follow-up data on the audit date were excluded to minimise potential misclassification errors.

### Outcome measures

The primary outcome was the prevalence of PHOP for the 17 indicators identified through a prior literature review and a Delphi consensus exercise (table 1).^26^ Experts involved in the Delphi survey included general practitioners, pharmacists, pain specialists, academic researchers and anaesthetists to determine agreement on the applicability of those indicators. The research team developed the operational definitions explicitly for use in the electronic health records based on the research purpose and recommendations from the Faculty of Pain Medicine (online supplemental material 1).^21^

**Table 1:** Definition of indicators of potentially hazardous opioid prescribing in primary care

No	Indicators	Denominator	Numerator
P1	Co-prescription of opioids with anti-epileptics to a patient with epilepsy	Epilepsy at least 3 months prior to AD	Antiepileptic prescription on the same day as opioid prescription, within 3 months of AD
P2	Co-prescription of opioids with antidepressants	Any opioid prescription in the 3 months prior to AD	Antidepressant prescription on same day as opioid prescription, within 3 months prior to AD
P3	Co-prescription of opioids with a benzodiazepine	Any opioid prescription in the 3 months prior to AD	Benzodiazepine prescription on same day as opioid prescription, within 3 months prior to AD
P4	Co-prescription of opioids with a gabapentinoid	Any opioid prescription in the 3 months prior to AD	Gabapentinoid prescription on same day as opioid prescription, within 3 months prior to AD
P5	Persistent prescription of opioids to a patient with dementia	Dementia records at least 6 months prior to AD	OPC >28 days within 6 months prior to AD
P6	Persistent prescription of opioids to a patient aged >65 years with recent records of falling	Aged over 65 years and has records for a fall between 6 and 18 months prior to AD	OPC >28 days within 6 months prior to AD
P7	Persistent prescription of opioids to a patient with a medical history of alcohol misuse, addiction, abuse or dependence	Alcohol misuse, addiction, abuse or dependence at least 6 months prior to AD	OPC >28 days within 6 months prior to AD
P8	Persistent prescription of opioids to a patient with hypothyroidism	Hypothyroidism at least 6 months prior to AD	OPC >28 days within 6 months prior to AD
P9	Persistent prescription of opioids to a patient with paralytic ileus	Paralytic ileus at least 6 months prior to AD	OPC >28 days within 6 months prior to AD
P10	Persistent prescription of opioids to a patient with COPD or asthma	COPD or asthma at least 6 months prior to AD	OPC >28 days within 6 months prior to AD
P11	Persistent prescription of opioids to a patient with myasthenia gravis	Myasthenia gravis at least 6 months prior to AD	OPC >28 days within 6 months prior to AD
P13	Persistent prescription of opioids to a patient with constipation without a concurrent laxative	OPC >28 days and has records for constipation within 6 months prior to AD	No laxative prescription following first opioid prescription in period of persistent coverage
P14	Prescription of codeine or morphine to a patient with severe renal impairment	Severe renal impairment or test result indicating eGFR <30 mL/min/173 m^2^ at least 3 months prior to AD	Codeine or morphine prescription within 3 months prior to AD
P15	Persistent prescription of opioids at a dose above 120 mg OMEQ per day	Any opioid prescription in the 6 months prior to AD	OPC >28 days with mean supplied dose >120 mg OMEQ dose within 6 months prior to AD
P16	Persistent prescription of opioids to a patient with at least moderate hepatic impairment	Moderate or severe hepatic impairment at least 6 months prior to AD	OPC >28 days within 6 months prior to AD
P17	Persistent prescription of tramadol, buprenorphine or oxycodone to a patient with ventricular tachycardia	Ventricular tachycardia at least 6 months prior to AD	Persistent tramadol, buprenorphine or oxycodone prescription (OPC >28 days) within 6 months prior to AD

AD, audit date; COPD, chronic obstructive pulmonary disease; eGFR, estimated glomerular filtration rate; OMEQ, oral morphine equivalent; OPC, opioid prescription day cover.

The prevalence of PHOP is a proportion and consists of a denominator and a numerator. The denominator included all patients with the potential to trigger this indicator due to existing prescribing or diagnosis records in an observed period specific to the indicator definition. The numerator included patients who actually triggered this indicator by receiving PHOP in an observed period. Those indicators were grouped into two composite indicators: co-medications (P1–P4) and elderly (P5–P6), with the denominator comprising all patients eligible for any indicator within the composite and counting patients only once in both the numerator and denominator.

Based on the British National Formulary, prescribed opioids are oral and transdermal formulations of opioid prescriptions, including buprenorphine, codeine, dextropropoxyphene, dihydrocodeine, fentanyl, hydromorphone, meptazinol, morphine, oxycodone, pethidine, tapentadol and tramadol.

### Covariates

Patient characteristics, including patients’ age, gender and the number of medications prescribed, as well as practice-level characteristics such as list size on the audit date, IMD quintile and regional location of the practice, which were associated with potential inappropriate prescribing in published studies, were examined.

The prescribing records from 1 January 2021 to 31 March 2021 were reviewed to assess the number of medications prescribed within 3 months before the audit date (opioids included) to indicate the severity of health conditions. The IMD serves as an area-level measure quantifying socioeconomic status at a low geographical level (averaging 1600 people) in the UK, based on multiple indices across seven domains, aggregated to maintain anonymity and reported in quintiles.

In addition, practices were classified into geographical regions by location, including Northwest, Northeast, Yorkshire and Humber, East Midlands, West Midlands, East of England, Southwest, South-Central, South East Coast and London.

### Data analysis and sensitivity analysis

The prevalence of PHOP was calculated by dividing the numerator by the denominator, with 95% CIs derived from a binomial distribution. An empty multilevel mixed-effects logistic regression model was used to quantify the variation in prevalence between practices, nesting patients within practices. Intraclass correlation coefficients (ICCs) were computed for each indicator to quantify the proportion of total variation attributable to differences in PHOP prevalence between practices,^30^ with higher ICC values indicating greater variability between practices compared with within them.

The reliability of an indicator, defined by the observed differences between practices that can be confidently attributed to true differences in the quality of prescribing, was calculated by the Spearman-Brown prophecy formulation for each practice.^31^ Reliability >0.7 is considered acceptable. The proportion of practices with reliability exceeding 0.7 was reported for each indicator,^27 32^ alongside the reliability of the practice, with the median patient count in the denominator.

Lastly, multilevel mixed-effects logistic regression models were applied to assess the characteristics associated with triggering any PHOP indicator, incorporating interaction terms between age categories and the number of medications prescribed. Results were presented as adjusted ORs (aORs) with 95% CIs. Based on published studies, characteristics with the potential to be associated with PHOP were included in the full regression model, and the results from the parsimonious models were presented after excluding non-significant variables.

## Results

### Prevalence of potentially hazardous opioid prescribing

The study cohort comprised 3 121 852 patients from 1358 general practices, with a median age of 56 (IQR 39, 72), of whom 44.86% were male. Overall, 361 505 of the 3 121 852 included patients (11.58%, 95% CI 11.54%, 11.62%) triggered at least one of the designated indicators (table 2). Among them, 667 732 patients had the potential to trigger a co-medication indicator (C1), and 261 148 patients (39.11%, 95% CI 38.99%, 39.23%) actually triggered this co-medication indicator. For indicators related to the elderly (C2), 138 088 patients with either dementia or a recent record of falling were identified, of which 21 306 patients (15.43%, 95% CI 15.24%, 15.62%) received persistent opioid prescriptions.

**Table 2:** Prevalence and variation of potentially hazardous opioid prescribing between practices in the primary care

No.	Indicators of potentially hazardous opioid prescribing	Numerator/Denominator	Prevalence (%)*	ICC	Rel	Rel>0.7
P1	Co-prescription of opioids with anti-epileptics to a patient with epilepsy	219/11 095	1.9 (1.7, 2.3)	0.03 (0.01, 0.30)	0.17	<1%
P2	Co-prescription of opioids with antidepressants	210 671/657 930	32.0 (31.9, 32.1)	0.02 (0.02, 0.02)	0.9	96%
P3	Co-prescription of opioids with a benzodiazepine	31 844/657 930	4.8 (4.8, 4.9)	0.05 (0.05, 0.06)	0.96	99%
P4	Co-prescription of opioids with a gabapentinoid	90 323/657 930	13.7 (13.7, 13.8)	0.02 (0.02, 0.03)	0.91	96%
P5	Persistent prescription of opioids to a patient with dementia	10 859/82 241	13.2 (13.0, 13.4)	0.02 (0.02, 0.03)	0.54	19%
P6	Persistent prescription of opioids to a patient aged >65 years with recent records of falling	12 479/67 935	18.4 (18.1, 18.7)	0.02 (0.01, 0.02)	0.39	8%
P7	Persistent prescription of opioids to a patient with a medical history of alcohol misuse, addiction, abuse or dependence	33 067/390 225	8.5 (8.4, 8.6)	0.08 (0.07, 0.09)	0.95	99%
P8	Persistent prescription of opioids to a patient with hypothyroidism	41 120/481 884	8.5 (8.5, 8.6)	0.07 (0.06, 0.07)	0.96	99%
P9	Persistent prescription of opioids to a patient with paralytic ileus	198/1711	11.6 (10.1, 13.2)	<0.01 (<0.01, <0.01)	<0.01	<1%
P10	Persistent prescription of opioids to a patient with COPD or asthma	114 571/1 626 040	7.1 (7.0, 7.1)	0.08 (0.07, 0.09)	0.99	100%
P11	Persistent prescription of opioids to a patient with myasthenia gravis	384/4030	9.5 (8.6, 10.5)	0.03 (<0.01, 0.28)	0.09	<1%
P12	Prescription of opioids to a patient with galactosaemia	91/841	10.8 (8.8, 13.1)	0.09 (0.01, 0.43)	0.09	<1%
P13	Persistent prescription of opioids to a patient with constipation without a concurrent laxative	2726/9155	29.8 (28.8, 30.7)	0.01 (<0.01, 0.04)	0.07	<1%
P14	Prescription of codeine or morphine to a patient with severe renal impairment	53 874/460 746	11.7 (11.6, 11.8)	0.03 (0.03, 0.04)	0.91	94%
P15	Persistent prescription of opioids at a dose above 120 mg OMEQ per day	26 938/824 396	3.3 (3.2, 3.3)	0.05 (0.05, 0.06)	0.97	100%
P16	Persistent prescription of opioids to a patient with at least moderate hepatic impairment	5409/38 417	14.1 (13.7, 14.4)	0.04 (0.03, 0.05)	0.49	8%
P17	Persistent prescription of tramadol, buprenorphine or oxycodone to a patient with ventricular tachycardia	264/8172	3.2 (2.9, 3.6)	0.10 (0.04, 0.23)	0.36	2%
C1	P1–P4	261 148/667 732	39.1 (39.0, 39.2)	0.02 (0.02, 0.02)	0.89	94%
C2	P5–P6	21 306/138 088	15.4 (15.2, 15.6)	0.02 (0.02, 0.02)	0.63	35%
Any	Any indicator	361 505/3 121 852	11.6 (11.5, 11.6)	0.07 (0.06, 0.07)	0.99	100%

* Prevalence was presented as percentage and 95% CI.

COPD, chronic obstructive pulmonary disease; ICC, intraclass correlation coefficient; OMEQ, oral morphine equivalent; Rel, reliability.

The prevalence of PHOP varied considerably across the 17 indicators, ranging from 1.97% to 32.02%. Notably, the most common PHOP in the general practices was the ‘co-prescription of opioids with antidepressants’. Additionally, 3.27% (95% CI 3.23, 3.31) of patients received opioid prescriptions exceeding 28 days at a dose above 120 mg OMEQ per day within the 6 months prior to the audit date.

### Variation and reliability in the prevalence of potentially hazardous opioid prescribing

Across the 1358 general practices, the prevalence of PHOP exhibited substantial variability, ranging from 0% (95% CI 0%, 4.62%) to 33.75% (95% CI 31.30%, 36.28%), with the 90th percentile at 17.88% (95% CI 16.39%, 19.44%). A significant number of practices, specifically 972 practices (71.6%), had the prevalence of PHOP over the 90th percentile for at least one of the 17 indicators. Among these, 557 practices (41.0%) had a prevalence of PHOP over the 90th percentile for more than one indicator.

The ICC for triggering any indicator was 0.07 (95% CI 0.06, 0.07). For the two composite indicators (co-medication and elderly), the ICCs were both 0.02 (table 2). Among the 17 individual indicators, the ICC ranged from <0.01 to 0.10, indicating that most indicators exhibited <10% of the variation attributed to differences between practices (table 2).

Indicators with high between-practice variation and reliability included persistent prescription of opioids to a patient with a history of alcohol use issues (P7) (ICC 0.08, 95% CI 0.07, 0.09), chronic obstructive pulmonary disease (COPD) or asthma (P10) (ICC 0.08, 95% CI 0.07, 0.09) and hypothyroidism (P8) (ICC 0.07, 95% CI 0.06, 0.07). Conversely, the ICC for indicators, such as co-medications (C1) (ICC 0.02, 95% CI 0.02, 0.02), prescription of codeine or morphine to a patient with severe renal impairment (P14) (ICC 0.03, 95% CI 0.03, 0.04) and persistent prescription of opioids at a dose above the 120 mg OMEQ per day (P15) (ICC 0.05, 95% CI 0.05, 0.06) indicated high reliability but less between-practice variation.

The reliability of triggering any one indicator was estimated at 0.99 for a practice with the median number of patients registered. The reliability of the two composite indicators was 0.89 (co-medications) and 0.63 (elderly), respectively. Eight of the 17 indicators had adequate reliability (>0.7) for a practice with the median number of patients registered. Notably, eight indicators achieved reliability >0.7 in over 90% of practices, but six indicators had reliability exceeding 0.7 in fewer than 5% of practices.

### Characteristics associated with potentially hazardous opioid prescribing

The prevalence of PHOP was notably higher in females (13.82%, 95% CI 13.77%, 13.87%), patients aged between 51 and 60 years (15.93%, 95% CI 15.83%, 16.04%) and those prescribed >10 medications (45.29%, 95% CI 45.09%, 45.48%). After accounting for all factors and the interaction between age and number of medications prescribed, factors associated with a higher risk of PHOP included: increasing age (aOR 1.74, 95% CI 1.55, 1.94 for those aged over 80) years and the number of medications prescribed (aOR 177.63, 95% CI 171.14, 184.38 for those with over 10 medications) (table 3).

**Table 3:** Characteristics associated with potentially hazardous opioid prescribing

Covariate and level (number at risk)	Prevalence (%)*	uOR (95% CI)	aOR (95% CI)
Gender			
Male (1 400 527)	8.8 (8.8, 8.9)	1	1
Female (1 721 325)	13.8 (13.8, 13.9)	1.66 (1.64, 1.67)	1.55 (1.54, 1.56)
Age			
18≤age≤50 (1 272 018)	6.5 (6.4, 6.5)	1	1
51≤age≤60 (523 957)	14.8 (14.7, 14.9)	2.42 (2.40, 2.45)	1.45 (1.38, 1.52)
61≤age≤70 (479 354)	15.9 (15.8, 16.0)	2.61 (2.58, 2.64)	1.32 (1.24, 1.41)
71≤age≤80 (471 324)	14.6 (14.5, 14.7)	2.36 (2.33, 2.38)	1.23 (1.13, 1.35)
81≤age (375 199)	15.1 (15.0, 15.2)	2.53 (2.50, 2.56)	1.74 (1.55, 1.94)
NoMP			
0–1 (1 265 669)	0.9 (0.8, 0.9)	1	1
2–4 (777 830)	7.8 (7.8, 7.9)	8.99 (8.80, 9.17)	12.53 (12.20, 12.88)
5–7 (519 664)	17.5 (17.4, 17.6)	22.17 (21.73, 22.62)	45.73 (44.46, 47.05)
8–10 (306 432)	27.5 (27.4, 27.7)	39.68 (38.89, 40.50)	89.63 (86.71, 92.66)
>10 (252 257)	45.3 (45.1, 45.5)	86.56 (84.83, 88.32)	177.63 (171.14, 184.38)
List size			
38–8376 (780 463)	12.7 (12.6, 12.8)	1	1
8376–12 639 (780 463)	12.2 (12.1, 12.3)	0.96 (0.90, 1.02)	NA
12 639–17 896 (780 463)	11.4 (11.3, 11.4)	0.88 (0.82, 0.94)	NA
17 896–128 425 (780 463)	10.1 (10.0, 10.2)	0.74 (0.69, 0.80)	NA
IMD			
1 (least deprived) (513 634)	9.0 (8.9, 9.0)	1	1
2 (498 509)	10.1 (10.0, 10.2)	1.15 (1.06, 1.25)	1.06 (1.01, 1.11)
3 (647 993)	10.7 (10.7, 10.8)	1.19 (1.10, 1.29)	1.09 (1.04, 1.14)
4 (690 638)	11.9 (11.8, 11.9)	1.33 (1.66, 1.94)	1.17 (1.12, 1.23)
5 (most deprived) (771 078)	14.7 (14.7, 14.8)	1.80 (1.66, 1.94)	1.28 (1.22, 1.35)
Region			
London (540 512)	7.7 (7.6, 7.7)	1	1
East of England (123 952)	9.0 (8.8, 9.1)	1.17 (1.03, 1.33)	1.29 (1.19, 1.40)
South Central (356 404)	9.7 (9.6, 9.8)	1.28 (1.17, 1.39)	1.35 (1.28, 1.43)
East Midlands (65 757)	10.6 (10.4, 10.8)	1.35 (1.17, 1.57)	1.39 (1.26, 1.53)
South East Coast (258 388)	10.9 (10.8, 11.0)	1.46 (1.33, 1.59)	1.46 (1.38, 1.55)
Southwest (392 221)	11.4 (11.3, 11.5)	1.55 (1.43, 1.68)	1.52 (1.45, 1.61)
West Midlands (538 955)	12.8 (12.8, 12.9)	1.80 (1.69, 1.92)	1.53 (1.46, 1.60)
Yorkshire/Humber (105 078)	13.8 (13.6, 14.0)	1.89 (1.66, 2.16)	1.57 (1.44, 1.71)
Northeast (116 481)	14.2 (14.0, 14.4)	2.11 (1.88, 2.37)	1.58 (1.46, 1.70)
Northwest (621 575)	15.1 (15.1, 15.2)	2.24 (2.10, 2.38)	1.73 (1.66, 1.81)

* Prevalence was presented as percentage and 95% CI.

aOR, adjusted OR; IMD, Index of Multiple Deprivation; NA, non-significant factors which were dropped from the model; NoMP, number of medications prescribed; uOR, unadjusted OR.

**Figure 1:**
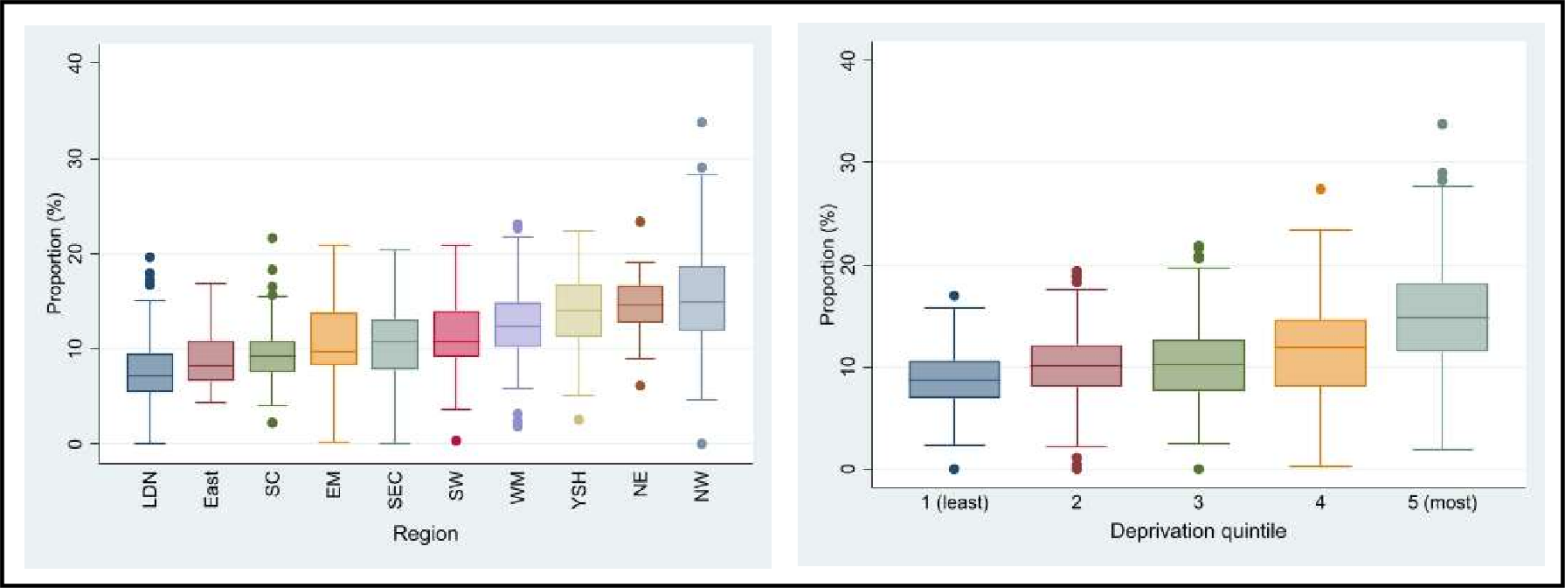
Prevalence of potentially hazardous opioid prescribing across general practices in different geographical regions and deprived areas. EM, East Midlands; LDN, London; NE, Northeast; NW, Northwest; SC, South-Central; SEC, South East Coast; SW, Southwest; WM, West Midlands; YSH, Yorkshire and Humber.

At the practice level, the prevalence of PHOP varied by IMD and geographic region. Patients registered in practices in the most deprived areas demonstrated a higher risk of PHOP (aOR 1.28, 95% CI 1.22, 1.34). Compared with those in London, patients registered in practices of other regions had higher risks for PHOP, with those in Southwest, West Midlands, Yorkshire/Humber, Northeast and Northwest being 50% more likely to receive PHOP, particularly those in the Northwest (aOR 1.73, 95% CI 1.66, 1.81) (table 3 and figure 1).

## Discussion

### Main findings

Among the patients at risk of triggering any PHOP indicator, approximately 10% met at least one of these indicators. Notably, co-medication indicators, particularly the co-prescription of opioids with antidepressants (32%) and gabapentinoids (13.7%), were common in patients already prescribed opioids. However, variability between general practices accounted for <10% of the differences in PHOP rates. Indicators such as persistent prescriptions for patients with alcohol use issues, hypothyroidism and COPD or asthma demonstrated greater reliability and higher between-practice variation. In particular, around 560 practices could have a higher prevalence for more than one PHOP indicator, highlighting important targets for future interventional studies, particularly in more deprived areas.

Conversely, indicators such as co-medication, the prescription of codeine or morphine for patients with severe renal impairment and persistent opioid prescriptions exceeding 120 mg OMEQ per day exhibited low ICCs and high variability within practices. Our findings suggested that patient characteristics, including age and the number of medications prescribed, significantly influence these indicators.

### Strengths and limitations

This study used a comprehensive list of prescribing indicators derived from two-round Delphi exercises with experts, ensuring face validity.^26^ Furthermore, the use of CPRD Aurum, which is representative of the English population regarding age, gender, geographic distribution and socioeconomic status, enhances the credibility of our findings.^28^ Some conditions, such as COPD or asthma, are included in the Quality and Outcomes Framework indicators and likely have well-documented diagnosis records within the CPRD.^33^ In addition, the link of the practice-level IMD quintile provides a comprehensive record of socioeconomic status in the neighbourhood area of the practice and facilitates the identification of targeted practices.

Despite these strengths, this study has limitations. There were limited numbers of patients with specified conditions, such as paralytic ileus and galactosaemia, which may challenge corresponding indicators in general practices. Moreover, while the CPRD Aurum database is representative of the geographic spread of the English population, it exclusively includes records from practices using the EMIS electronic patient record system software.^28^ Consequently, the results and conclusions may not be generalised to practices using alternative electronic patient record systems due to differences in recorded clinical data.^34^

In addition, because each indicator targets different clinical focuses, and the denominators include patients with diverse characteristics, we selected and adjusted for characteristics supported by existing evidence to identify key factors for targeting future interventions. Further studies could incorporate additional covariates, such as comorbidities, when applying these indicators in specific clinical contexts. Aligned with our research purpose, we used an established and widely used definition of persistent opioid use (28 days), but we acknowledge that this is a pragmatic choice and may incorrectly classify some patients.

### Comparison with published studies

To our knowledge, this is the first study to investigate the variation in prevalence of multiple indicators of PHOP within UK primary care. In contrast to a previous study that identified the prevalence of persistent opioid prescribing and the associated characteristics,^35^ our study quantified a broad spectrum of PHOP types. Our findings align with existing literature linking persistent opioid prescribing to deprived socioeconomic status,^36 37^ indicating that deprived socioeconomic status is associated not just with persistent use but with a range of hazardous prescribing practices.

The ICC value of triggering any PHOP indicator is 0.07 in our study, which is higher than the ICC value for triggering any potentially hazardous prescribing of other medications reported in a published study that also used primary care data from England.^27 32^ However, we found that most opioid prescribing indicators had ICCs below 0.1, which is lower than most of the ICC values for safety indicators of potentially hazardous prescribing of other medications.^27 32^ This may reflect the cluster effect due to local opioid prescribing guidelines or policies,^38 39^ which could differ significantly from those applicable to other medications. Further intervention plans should be aware of the potential cluster effect when designing a protocol to optimise opioid prescribing.

### Implications and further recommendations

Indicators with high reliability and larger patient denominators, such as co-medications (P2–P4), high-dose opioid prescribing (P15) and persistent opioid prescribing for patients with alcohol use issues (P7), hypothyroidism (P8) or COPD or asthma (P10), are likely to be more feasible for targeting as part of interventions, considering their prevalence and variability. A higher ICC value indicates a greater variation between practices and can be attributed to several factors, including healthcare practitioners’ clinical knowledge, prescriber training and adherence to prescribing guidelines.^36 37^ Although prescribing is commonly recorded systematically in the electronic system, inconsistent documentation of specific disease conditions may also contribute to variations in the prevalence of PHOP.^33^ Therefore, further intervention plans should be cautious when implementing and monitoring those indicators in general practices.

In addition to the ICC value, other criteria, such as indicators that show the poorest scores, potential risk of adverse events and the prevalence, could be considered when implementing those indicators in the interventions. Our findings further indicate that compared with patients registered in practices in the less deprived areas, patients registered at practices located in the deprived areas were more likely to experience PHOP. Deep-end practices in Scotland have between 50% and 90% of their registered patients drawn from the 15% most deprived areas, with the prevalence of health problems nearly tripling across the socioeconomic spectrum. Supporting these practices with tailored strategies and resources will be essential for improving prescribing safety and addressing health inequities.^40^ According to the 2011 Health Survey for England, lower household income is associated with a higher prevalence of chronic pain conditions, and deprived socioeconomic status, including unemployment, was linked to higher rates of opioid prescribing.^39 41 42^

In addition, although non-pharmacological interventions, such as cognitive behavioural therapy, were preferred for patients with chronic pain, it is challenging to access non-pharmacological interventions for patients in deprived areas due to an imbalance in healthcare accessibility.^43^ Implementing more reliable indicators (with high reliability and ICC values), such as persistent opioid prescribing for patients with alcohol use issues, hypothyroidism, COPD or asthma, into interventions launched at primary care may provide an effective strategy to reduce hazardous opioid prescribing. For example, a pharmacist-led electronic audit and feedback intervention system resulted in a 40% reduction in (non-opioid) inappropriate prescribing in general practice in Salford, UK.^44 45^ Further research could evaluate the clinical and cost-effectiveness of implementing these specific opioid prescribing safety indicators in future work. The National Health Service Business Services Authority also developed the opioid prescribing comparators dashboard to monitor the number and duration of opioid prescribing to support practice in primary care.^46^ However, the feasibility and effectiveness of implementing those opioid prescribing indicators into the current intervention plan were still unclear. Further research should focus on integrating these indicators into interventions and evaluating their impact on prescribing practices.

For indicators with high reliability but lower ICC values, interventions should specifically target patients with identified risk factors to mitigate PHOP. This is particularly relevant for indicators associated with co-prescribing and those indicating high doses of opioids (above 120 mg OMEQ per day), commonly observed in patients with chronic non-cancer pain in primary care,^14 15^ further emphasising the need for effective strategies to optimise chronic pain management.^2^

Indicators with low reliability suggest that many practices do not have sufficient patient numbers to confidently attribute the variability of these indicators to differences between practices. In contrast to individual indicators, the composite indicators related to co-medications have adequate reliability, indicating that monitoring the co-prescription of opioids alongside antidepressants, gabapentinoids and benzodiazepines might be a more feasible approach for general practices. Moreover, the identification of practices exceeding the 90th percentile for any indicator on multiple occasions highlights the need for targeted monitoring of those practices, which could efficiently reduce PHOP.

## Conclusion

Approximately 10% of patients with the potential to trigger any PHOP indicator met at least one prescribing indicator in primary care. However, the variation between practices explains <10% of the observed differences, likely reflecting the influence of local opioid prescribing guidance and policies. Further studies are warranted to explore the feasibility of integrating opioid prescribing indicators, including persistent opioid prescribing for patients with alcohol use issues, hypothyroidism, COPD or asthma, into interventions, especially for patients with chronic non-cancer pain in more deprived areas.

## Supplementary material

10.1136/bmjqs-2025-018794 online supplemental file 1

## Data Availability

Data are available on reasonable request. Data may be obtained from a third party and are not publicly available.
