# Supplementary material for: Examining variations in the prevalence of hazardous opioid prescribing across general practices in England: a cross-sectional study
Source: BMJ Qual Saf. 2025 Aug 5;35(8):e018794. doi: 10.1136/bmjqs-2025-018794 (PMC13479247; doi:10.1136/bmjqs-2025-018794)
Supplement: Supplementary file 1 [file bmjqs-35-8-s001.docx]

Supplemental material 1.	Link to the medcodes and prodcodes used in this study

<https://github.com/TCCNYCU/Hazardous-opioid-prescribing/blob/dfe40f8f7ecadcfa8029ce578155d5f8dc3af0d4/250706_Supplemental%20material%201.xlsx>
